# Effectiveness of inpatient versus outpatient rehabilitation following total knee arthroplasty on clinical and functional outcomes in Germany: a non-randomized clinical trial

**DOI:** 10.1186/s13102-026-01735-4

**Published:** 2026-05-23

**Authors:** Anett Mau-Moeller, Nassim El-Aarid, Martin Behrens, Tino Stöckel, Philipp Bergschmidt, Rainer Bader, Stephan Tohtz, Wolfram Mittelmeier, Robert Jacksteit

**Affiliations:** 1 Department of Orthopaedics, University Medical Center Rostock, Rostock, Germany; 2 Department of Ageing of Individuals and Society, University of Rostock, Rostock, Germany; 3 University of Applied Sciences for Sport and Management Potsdam, Potsdam, Germany; 4 Department of Orthopaedics and Traumatology, Klinikum Südstadt, Rostock, Germany; 5 Department of Orthopaedics and Traumatology, HELIOS Klinikum Emil von Behring GmbH, Berlin, Germany

**Keywords:** Osteoarthritis, Knee endoprosthesis, Physical activity, Timed up and go test, Stair climbing test, Joint position sense, Gait analysis, Structural dimensional analysis of mental representation (SDA-M), Motor performance

## Abstract

**Background:**

Rehabilitation models following total knee arthroplasty (TKA) vary internationally. The German healthcare system typically involves an initial hospital stay of 8–10 days, followed by a 3-week intensive rehabilitation program, which is delivered in either an inpatient or outpatient setting. Only a few studies have evaluated the outcomes of these two models on functional recovery in TKA patients. Therefore, this study compared the effectiveness of inpatient versus outpatient rehabilitation on clinical and functional outcomes in a German TKA cohort.

**Methods:**

This multicenter, prospective, non-randomized, active-controlled, investigator- and outcome assessor-blinded clinical trial allocated patients with primary TKA based on their preference to either a 3-week inpatient (*n* = 26) or outpatient (*n* = 16) rehabilitation program. Measurements were conducted at two time points: pre-test (one day prior to discharge from acute care hospital; pre-rehabilitation) and post-test (three months following TKA). The primary outcome, step count, was measured using 7-day activPAL™ accelerometery during the first week of inpatient and outpatient rehabilitation, respectively. The clinical outcomes included knee pain, swelling, and range of motion (ROM), whereas the functional outcomes included step count, sit-to-stand transitions, timed up and go (TUG) and stair climbing (SC) performance, joint position sense (JPS), and gait performance. Between-group differences were analysed using analysis of covariance, adjusting for baseline values (where available), age, BMI, sex, hospital, and the total number of interventions during rehabilitation.

**Results:**

At pre-test, the outpatient group had a significantly higher covariate-adjusted step count compared to the inpatient group (*P* < 0.001; *η*_*p*_^*2*^ = 0.338; +73.8%). At post-test, the outpatient group demonstrated significantly better JPS, with a lower mean absolute error at both 30° (*P* < 0.001; *η*_*p*_^*2*^ = 0.366; − 64.6%) and 50° (*P* = 0.010; *η*_*p*_^*2*^ = 0.180; − 47.1%) of knee flexion. The outpatient group also showed superior SC performance, completing the task 5.3 s faster (*P* = 0.004; *η*_*p*_^*2*^ = 0.218; − 23.7%). No significant between-group differences were found for knee pain, swelling, ROM, TUG performance, and gait performance (post-hoc power *β* < 0.50, suggesting a high risk of type II error).

**Conclusions:**

Results of the study indicate that outpatient rehabilitation promoted higher early physical activity levels (step count) and superior functional outcomes (JPS and SC performance). These preliminary findings must be interpreted with caution due to study limitations, including the non-randomized design and a small sample size.

**Trial registration:**

ClinicalTrials.gov, NCT02120313. Registered 23 April 2014.

**Level of evidence:**

Level 3 (OCEBM Levels of Evidence Working Group). "The Oxford Levels of Evidence 2". Oxford Centre for Evidence-Based Medicine. http://www.cebm.net/index.aspx?o=5653

**Supplementary Information:**

The online version contains supplementary material available at 10.1186/s13102-026-01735-4.

## Introduction

Total knee arthroplasty (TKA) is a widely performed and highly effective surgical intervention for individuals diagnosed with end-stage knee osteoarthritis (KOA), yielding significant improvements in pain, function, and quality of life. Postoperative rehabilitation is crucial for optimizing these and other outcomes including the recovery of strength, improving physical function, and enabling a return to daily activities [1, 2].

The rehabilitation approach after TKA varies considerably across countries, influenced by diverse healthcare systems, economic pressures, and cultural preferences. A robust body of evidence from systematic reviews with meta-analyses considering different countries consistently suggests that home-based and outpatient rehabilitation produce clinical outcomes that are largely comparable to those of traditional inpatient rehabilitation programs [3–6].

However, Germany employs a distinctive and comprehensive rehabilitation model, characterized by a relatively long acute hospital stay (8–10 days), followed by a 3-week rehabilitation period [7]. The right to “preference and choice” also allows patients to select their preferred rehabilitation facility including inpatient and outpatient options [8]. Despite this choice, a substantial majority of patients with TKA (85.4%) undergo a 3-week inpatient program in Germany [8]. This extensive rehabilitation pathway is supported and covered by Germany’s social insurance system which covers rehabilitation costs, and a societal preference for thorough, structured, and professionally supervised care. This rehabilitation model, however, stands in contrast to a strong international trend towards shorter hospital stays and a quicker transition to outpatient or home-based rehabilitation [3–6]. This global shift underscores the need to critically evaluate the traditional German rehabilitation model.

While previous German studies on mixed orthopaedic populations (e.g., end-stage KOA, degenerative spine, other joint replacements, chronic pain) suggest that outpatient rehabilitation offers comparable quality [9–14] and is more cost-effective [9, 10, 15], there is a lack of evidence specifically comparing inpatient and outpatient rehabilitation for patients with TKA focussing on functional outcomes. This study aimed to address this gap and analysed the effectiveness of inpatient and outpatient rehabilitation on clinical (i.e., pain, swelling, active and passive maximal knee flexion and extension range of motion) and functional outcomes (i.e., step count and number of sit-to-stand transitions over 7 days, timed up and go performance, stair climbing performance, joint position sense, gait performance, and long-term memory representation of the gait) in a German TKA cohort. It was hypothesized that the rehabilitation setting (inpatient versus outpatient) would result in differences regarding physical activity levels (measured by step count), clinical outcomes, and functional performance.

## Materials and methods

### Study design and ethics statement

The study was designed as a multicenter (three hospitals), two-armed, parallel-group, non-randomized, active-controlled, investigator- and outcome assessor-blinded clinical trial. It is classified as active-controlled, because it compared two established, active therapeutic interventions, i.e., inpatient rehabilitation (the traditional standard of care in Germany) versus outpatient rehabilitation (the comparator intervention). The inclusion of a control group receiving no intervention was not possible for ethical reasons, given that post-operative rehabilitation is the medical standard of care for restoring function after TKA in Germany. Withholding a treatment would be contrary to the interests of the patients and violate the ethical principles of the Declaration of Helsinki.

Randomization was not feasible due to the legal right to “preference and choice” in the German healthcare system. Attempting to randomize patients is often rejected by individuals with strong rehabilitation preferences. Forcing a highly motivated patient into inpatient care, or a less mobile patient into outpatient care, would likely lead to high dropout rates and ethical concerns. Therefore, completing the study required an extended period of time because it was difficult to recruit patients who had opted for outpatient rehabilitation. In Germany, outpatient care is only chosen by 14.6% of patients [8].

The study protocol received approval from the Ethical Review Committee of the Rostock University Medical Center (A 2014-0027) and was conducted in accordance with the Declaration of Helsinki. All participants provided written informed consent before enrollment, and the trial is registered with ClinicalTrials.gov (NCT02120313). This study was supported by the Stiftung Oskar-Helene-Heim (funding grant).

### Participants

Participants eligible for the study were 50 to 80 years of age and scheduled to undergo a primary TKA for KOA. This study employed a non-randomized design based on patient preference for either inpatient or outpatient rehabilitation. There are no preliminary studies on the comparison of both interventions with respect to the primary outcome in order to adequately calculate a sample size. Assuming a large effect (Cohen’s *f* = 0.40) with a two-sided significance of 0.050 and a power of 0.80, a total of 52 patients (26 patients in each group) would be needed.

The exclusion criteria were a body mass index (BMI) > 40 kg/m² (to reduce confounding effects from obesity-related mobility limitations), musculoskeletal disorders other than KOA (to ensure homogeneity), neurological disorders or metabolic bone disease (which could affect recovery), pain or restrictions precluding participation in physical performance tests (to ensure safety/validity), and contralateral TKA or hip replacement within the previous year. The demographic and clinical characteristics of the participants are provided in Table 1.

**Table 1:** Demographic and clinical characteristics of patients

	Descriptive statistics: Mean (standard deviation) or number (%)		Inferential statistics	
	Inpatient group (*n* = 26)	Outpatient group (*n* = 16)	Statistical test	*P*
Age (yrs)	71.9 (7.4)	68.8 (7.7)	Mann-Whitney-U-test	0.218
Weight (kg)	87.5 (12.5)	91.4 (15.7)	Unpaired Student’s t-test	0.380
Height (m)	1.70 (0.08)	1.71 (0.08)	Unpaired Student’s t-test	0.831
Body mass index (kg/m^2^)	30.2 (3.6)	31.4 (5.3)	Unpaired Student’s t-test	0.441
Sex (males)	11.0 (42.3%)	8.0 (50.0%)	Fisher’s exact test	0.753
Operated leg (right leg)	13.0 (50.0%)	3.0 (18.8%)	Fisher’s exact test	0.056
Mini-Mental State Examination	28.7 (1.4)	29.0 (1.3)	Mann-Whitney-U-test	0.503
Pre-test (postoperative day)	9.1 (0.4)	8.9 (0.3)	Mann-Whitney-U-test	0.150
Post-test (postoperative day)	92.9 (8.2)	93.2 (6.7)	Mann-Whitney-U-test	0.541
Length of hospital stay (day)	10.5 (1.7)	10.3 (1.9)	Mann-Whitney-U-test	0.495
Number of Comorbidities (ICD-Codes)			Fisher’s exact test	
B00-B99	1.0 (3.8%)	0.0 (0.0%)		1.000
C00-D48	6.0 (23.1%)	3.0 (18.8%)		1.000
D50-D90	2.0 (7.7%)	0.0 (0.0%)		0.517
E00-E90	10.0 (38.5%)	6.0 (37.5%)		1.000
F00-F99	1.0 (3.8%)	3.0 (18.8%)		0.146
G00-G99	3.0 (11.5%)	3.0 (18.8%)		0.658
H00-H59	1.0 (3.8%)	0.0 (0.0%)		1.000
H59-H99	0.0 (0.0%)	0.0 (0.0%)		-
I00-I99	23.0 (88.5%)	13.0 (81.3%)		0.658
J00-J99	5.0 (19.2%)	2.0 (12.5%)		0.690
K00-K93	3.0 (11.5%)	3.0 (18.8%)		0.658
M00-M99	15.0 (57.7%)	9.0 (56.3%)		1.000
N00-N99	1.0 (3.8%)	1.0 (6.3%)		1.000
R00-R99	1.0 (3.8%)	0.0 (0.0%)		1.000
Z00-Z99	2.0 (7.7%)	1.0 (6.3%)		1.000
Number of operations per surgeon			Pearson chi-squared test	0.014*
Surgeon 1	8.0 (30.8%)	0.0 (0.0%)		
Surgeon 2	3.0 (11.5%)	2.0 (12.5%)		
Surgeon 3	5.0 (19.2%)	0.0 (0.0%)		
Surgeon 4	6.0 (23.1%)	10.0 (62.5%)		
Surgeon 5	4.0 (15.4%)	4.0 (25.0%)		
Number of operations per hospital			Pearson chi-squared test	0.003*
Hospital 1	17.0 (65.4%)	2.0 (12.5%)		
Hospital 2	5.0 (19.2%)	10.0 (62.5%)		
Hospital 3	4.0 (15.4%)	4.0 (25.0%)		

*ICD:* International Statistical Classification of Diseases and Related Health Problems, *P: *Probability value (* *P* ≤ 0.050)

### Surgical procedure, pain management, and physiotherapy

All patients underwent a standardized surgical procedure performed by experienced surgeons involving the implantation of a bicondylar total knee endoprosthesis (Hospitals 1 and 3: e.motion^®^. Braun Melsungen AG, Melsungen, Germany; Hospital 2: Gemini^®^ SL^®^, Waldemar Link GmbH & Co. KG, Hamburg, Germany) using Payr’s approach. All types of implants were non-constrained surface replacement systems consisting of cemented metallic femoral and tibial components and equipped with ultrahigh-molecular weight polyethylene liners. Smoothening of the lateral patella facet, denervation, and soft-tissue balancing were carried out until perfect positioning of the implant components was achieved with respect to biomechanical aspects. Both the femoral and the tibial components were fixed with bone cement made of polymethylmethacrylate (PMMA). Postoperative pain was managed with either pain-adapted medical analgesia or continuous epidural or femoral nerve analgesia.

Full weight-bearing with crutches (four-point gait) was initiated on the second postoperative day. A standardized in-hospital physiotherapy program was commenced on the first postoperative day, consisting of daily 20–30-min sessions of active and passive range of motion exercises, quadriceps strengthening, and training for activities of daily living (e.g., transfer from bed to chair, transition from sitting to standing, walking, and climbing stairs). One unit of intervention is defined as a single, scheduled therapeutic session, typically lasting 20–60 min (e.g., one session of gait training or one application of continuous passive motion).

Exercise intensity was gradually increased according to pain and tolerance. Furthermore, patients received two (hospitals 1 and 2) or one (hospital 3) 30-min continuous passive motion (CPM) applications each day from the second postoperative day until 1 day prior to discharge. Patients were discharged once they were medically stable and able to demonstrate at least 90° of knee flexion and independent mobility for basic self-care (see Table 1 for length of hospital stay).

### Experimental design and procedures

**Fig. 1:**
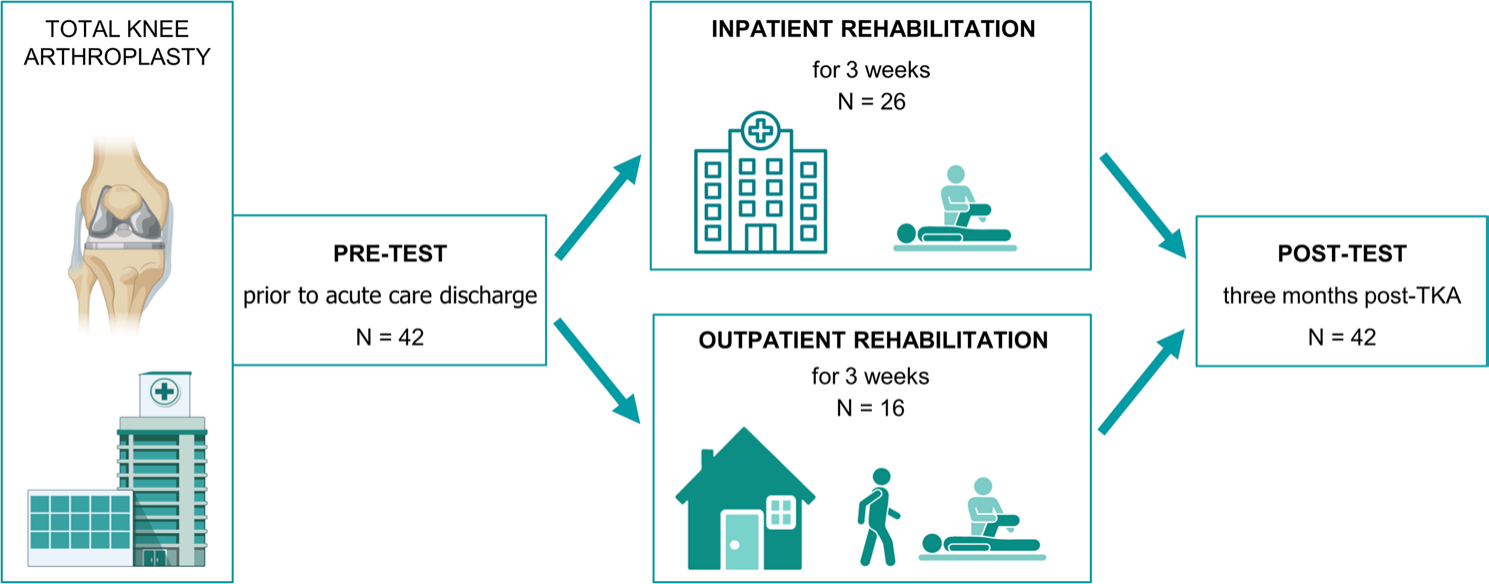
Flow diagram illustrating the study design, assessment time points, and interventions. Measurements were conducted at two time points: pre-test (one day before discharge from acute care hospital, i.e. pre-rehabilitation) and post-test (3 months post-TKA). Physical activity, the primary outcome, was continuously measured for 7 days at pre-test (coinciding with the first week of rehabilitation) and at post-test. Patients were allocated to a 3-week rehabilitation program based on their stated preference: (1) inpatient rehabilitation (continuous 3-week stay at a rehabilitation clinic) or (2) outpatient rehabilitation (daily physiotherapy sessions at an outpatient center while maintaining residence at home)

Measurements were performed at two time points: pre-test (conducted one day before discharge from acute care hospital, i.e., pre-rehabilitation) and post-test (conducted 3 months following TKA) (Fig. 1). The primary outcome, step count, was measured over a continuous 7-day period at pre-test (which coincided with the first week of rehabilitation) and at post-test.

Following discharge from the acute hospital, eligible patients were allocated to one of two 3-week rehabilitation programs based on their stated preference:

1.  Inpatient rehabilitation: Participants were admitted to a single designated rehabilitation clinic for a continuous 3-week period.
2.  Outpatient rehabilitation: Participants lived at home and attended daily therapy sessions at one of two independent outpatient rehabilitation centers.

Data were collected by two independent, blinded investigators.

### Clinical and functional outcomes

**Fig. 2:**
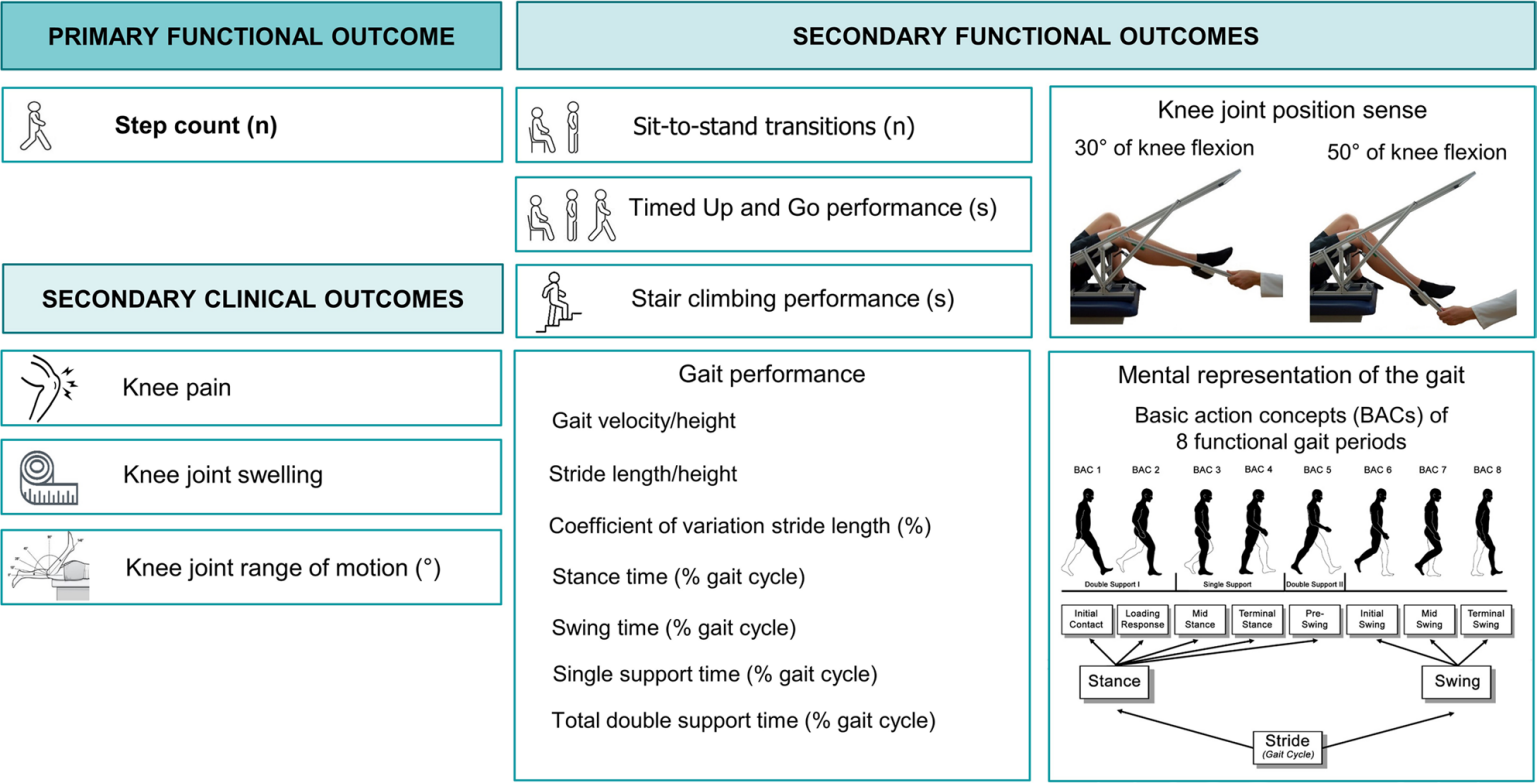
Overview of clinical and functional outcomes

The outcomes included knee-specific clinical parameters (i.e., pain, swelling, active and passive maximal knee flexion and extension range of motion) as well as key indicators of functional recovery (i.e., step count and number of sit-to-stand transitions over 7 days, timed up and go performance, stair climbing performance, joint position sense at 30° and 50° of knee flexion, gait performance, and long-term memory representation of the gait) (Fig. 2).

#### Primary functional outcome

Step count, the primary outcome, was measured over a continuous 7-day period using activPAL™ uniaxial accelerometers (PAL Technologies Ltd., Glasgow, UK) [16]. The number of sit-to-stand transitions was also calculated from the same data set as a secondary outcome. The sensor (35 × 63 × 7 mm; 20 g) was attached according to the manufacturer’s instructions anteriorly in the middle of the thigh of the unaffected leg at the pre-test assessment (during the first week of rehabilitation).

This placement at pre-test was chosen to (1) avoid interference from clinical mobilization of the operated limb and (2) minimize measurement bias caused by pain-related compensatory movements, thereby ensuring a more accurate assessment of baseline physical activity. At the post-test, the sensor was attached to the right thigh for all participants to provide a standardized placement. This transition is supported by evidence that step-count detection via thigh-worn uniaxial accelerometers is side-invariant in populations with stable mobility levels. Previous validation studies have demonstrated high test-retest reliability for the activPAL™ device [16] and excellent inter-device agreement (*intraclass correlation coefficient* ≥ 0.99) during simultaneous bilateral placement, regardless of the side of attachment [17]. Given that gait patterns typically stabilize and asymmetries resolve by three months post-TKA [18], this standardized placement ensures comparability without introducing measurement bias.

The detailed methodology for the use of these physical activity monitors in this patient population has been previously described [18].

#### Secondary clinical outcomes

##### Knee pain, swelling, and range of motion

Knee pain was evaluated using a visual analogue scale with a 0–100 mm range. Knee joint swelling was determined by measuring the circumference of the knee 1 cm above the superior patella border using a tape measure, a method that has demonstrated high intra- and intersession reliability in this patient population. Active and passive maximal knee flexion and extension range of motion were measured with a standard digital long-arm goniometer. These standardized procedures have been detailed previously [18].

#### Secondary functional outcome measures

##### Timed up and go and stair climbing performance

Functional performance was assessed using the timed up and go and stair climbing tests. For the timed up and go test, patients were instructed to rise from an armchair, walk 3 m, turn, return, and sit back down, with the time measured by a stopwatch. The stair climbing test required patients to ascend and descend an eight-step staircase as quickly as possible, with the time recorded via stopwatch. These methods have been previously described in detail [18].

#### Knee joint position sense

Proprioceptive accuracy was determined by assessing knee joint position sense at 30° and 50° of knee flexion, with accuracy defined as the mean absolute error between the target and reproduced knee angles. The 30° and 50° angles were specifically selected as they represent functionally relevant joint angles [19, 20]. The 30° angle is highly correlated with the proprioceptive feedback required for stability during the stance phase and regular walking. The 50° angle represents the midpoint of knee flexion, which is critical for performing high-demand physical tasks such as stair climbing. The detailed methodology for this measurement, which involves a custom-made splint and a twin-axis electrogoniometer, has been described in our prior publication [20].

#### Gait performance and mental representation of the human gait in long-term memory

Gait performance was assessed using the OptoGait floor-based photocell system (Microgate, Bolzano, Italy), which measured two spatio-temporal (gait velocity and stride length) and four temporophasic (stance time, swing time, single support time, and total double support time as a percentage of the gait cycle) parameters. Furthermore, the coefficient of variation (CV) of stride length (CV = [standard deviation / mean] x 100) was determined as a measure of gait variability and an indicator of falling risk [21].

The mental representation of the human gait in long-term memory was assessed via the structural dimensional analysis of mental representation (SDA-M). This method uses a hierarchical cluster analysis based on the perceived relationships between basic action concepts of the human gait (Fig. 2). The full methodology for both the gait performance and SDA-M assessments has been previously published in studies that explored the effects of aging, TKA, as weel as knee and hip osteoarthritis on gait [22–24].

### Statistical analyses

Descriptive statistics are reported as raw means and standard deviations on the original scale for all outcomes.

Data distribution was assessed using the Shapiro‒Wilk *W* test. Between-group differences in baseline characteristics were analysed using parametric or nonparametric tests (Fisher’s exact test, unpaired Student’s *t* test, Pearson’s chi-squared test, Mann‒Whitney *U* test; Table 1).

Primary analysis of outcomes was performed using analysis of covariance (ANCOVA) with Holm-Sidak post-hoc correction. Two distinct models were applied depending on the availability of baseline data:

ANCOVA-Model 1: For parameters measured primarily at one time point or representing independent activity levels (e.g., step count, sit-to-stand transitions, and gait performance; Table 3), the model controlled for age, BMI, sex, hospital, and the total number of interventions.

ANCOVA-Model 2: For clinical and functional outcomes with both pre- and post-test assessments (e.g., knee pain, joint swelling, range of motion, timed up and go performance, stair climbing performance, joint position sense, and lambda for mental representation; Table 4), the model additionally adjusted for the respective baseline (pre-test) value.

Inclusion of ‘hospital’ as a covariate served as an adjustment for baseline imbalances in surgical characteristics, including implant types, standardized surgical approaches, and operation volumes per hospital and surgeon (Table 1). Similarly, entering the ‘total number of interventions’ into the model controlled for variations in therapeutic interventions during the rehabilitation period (Table 2).

**Table 2:** Number of interventions during 3 weeks of inpatient and outpatient rehabilitation. Values for the inpatient group represent the fixed numbers from the standardized clinical protocol. Values for the outpatient group are presented as means (standard deviations), representing the average of two independent outpatient rehabilitation centers to allow for a direct comparison

	Inpatient group (*n* = 26)	Outpatient group (*n* = 16)	Subgroups: outpatient rehabilitation	
	Fixed protocol	Mean (standard deviation)	Center 1 (*n* = 12)	Center 2 (*n* = 4)
Physiotherapy knee (individual therapy)	10.0	8.3 (1.3)	9.0	6.0
Physiotherapy knee (group therapy)	9.0	8.8 (0.4)	9.0	8.0
Gait training	7.0	8.3 (1.3)	9.0	6.0
Aqua exercise	6.0	8.5 (0.9)	9.0	7.0
Bicycle ergometer training	7.0	9.8 (4.0)	12.0	3.0
Continuous passive motion	7.0	14.0 (1.8)	15.0	11.0
Medical training therapy	6.0	9.0 (0.0)	9.0	9.0
Manual lymphatic drainage	13.0	8.5 (0.9)	9.0	7.0
Training courses for patients	5.0	3.5 (0.9)	3.0	5.0
Physical therapy (incl. sling exercise training and traction treatment)	13.0	4.8 (1.3)	4.0	7.0
Total number of interventions	83.0	83.3	88.0	69.0

For non-normally distributed data, inferential statistics (*P*,* F*, and *η*_*p*_^*2*^) were calculated from the log-transformed (*Log*10) or reverse-transformed (1/x) data. To ensure mathematical coherence between the *P*-values, adjusted mean differences (MD), and 95% confidence intervals (CI) for these variables are reported on the respective transformed scale in all tables and figures. Clinical improvements are detailed in the text and figure as percentage differences. For log-transformed variables, percentage differences were calculated by back-transforming the covariate-adjusted MD (10^Adjusted MD^-1). For reverse-transformed stair climbing performance, percentages were calculated based on back-transformed adjusted means to reflect the improvement in seconds.

The level of significance was set at *P* ≤ 0.050. To contextualize the findings beyond the *P*-value, effect size partial eta squared (*η*_*p*_^*2*^) was calculated using G*Power 3.1 software, where *η*_*p*_^*2*^ ≥ 0.01 represents a small effect, *η*_*p*_^*2*^ ≥ 0.06 a medium effect, and *η*_*p*_^*2*^ ≥ 0.14 a large effect [25]. Post-hoc power (*β*) was calculated from observed effect size for all primary and secondary outcomes.

Partial Spearman’s rank correlations (*r*_*s*_) were used to assess relationships between functional outcomes at post-test (i.e., step count, sit-to-stand transitions, timed up and go performance, stair climbing performance, knee pain, and active knee flexion), controlled for age, BMI, sex, hospital, and number of interventions. Correlations were calculated using the pooled sample (*n* = 42); exploratory within-group analyses indicated that correlation patterns were generally consistent across both groups. The correlation was interpreted as weak (|*r*_*s*_| ≥ 0.1), moderate (|*r*_*s*_| ≥ 0.3) or strong (|*r*_*s*_| ≥ 0.5) [25].

Statistical analyses were performed using SPSS statistical package 22.0 (SPSS Inc., Chicago, IL, USA).

## Results

A total of 42 patients were recruited and completed the study. The target for inpatient rehabilitation was met (*n* = 26), but recruitment for the outpatient group was terminated prematurely due to the expiration of project funding, resulting in a smaller cohort (*n* = 16). All patients received the allocated intervention, and no adverse events were reported. There were no lost to follow-up, and all 42 participants (*n* = 26 inpatient, *n* = 16 outpatient) completed the post-test assessments.

No significant between-group differences were found for most demographic and clinical subject characteristics, except for the variables operated side or the number of operations per surgeon and per hospital (Table 1). The content and total volume of physiotherapeutic interventions were comparable between the groups, as detailed in Table 2.

### Primary functional outcome

**Fig. 3:**
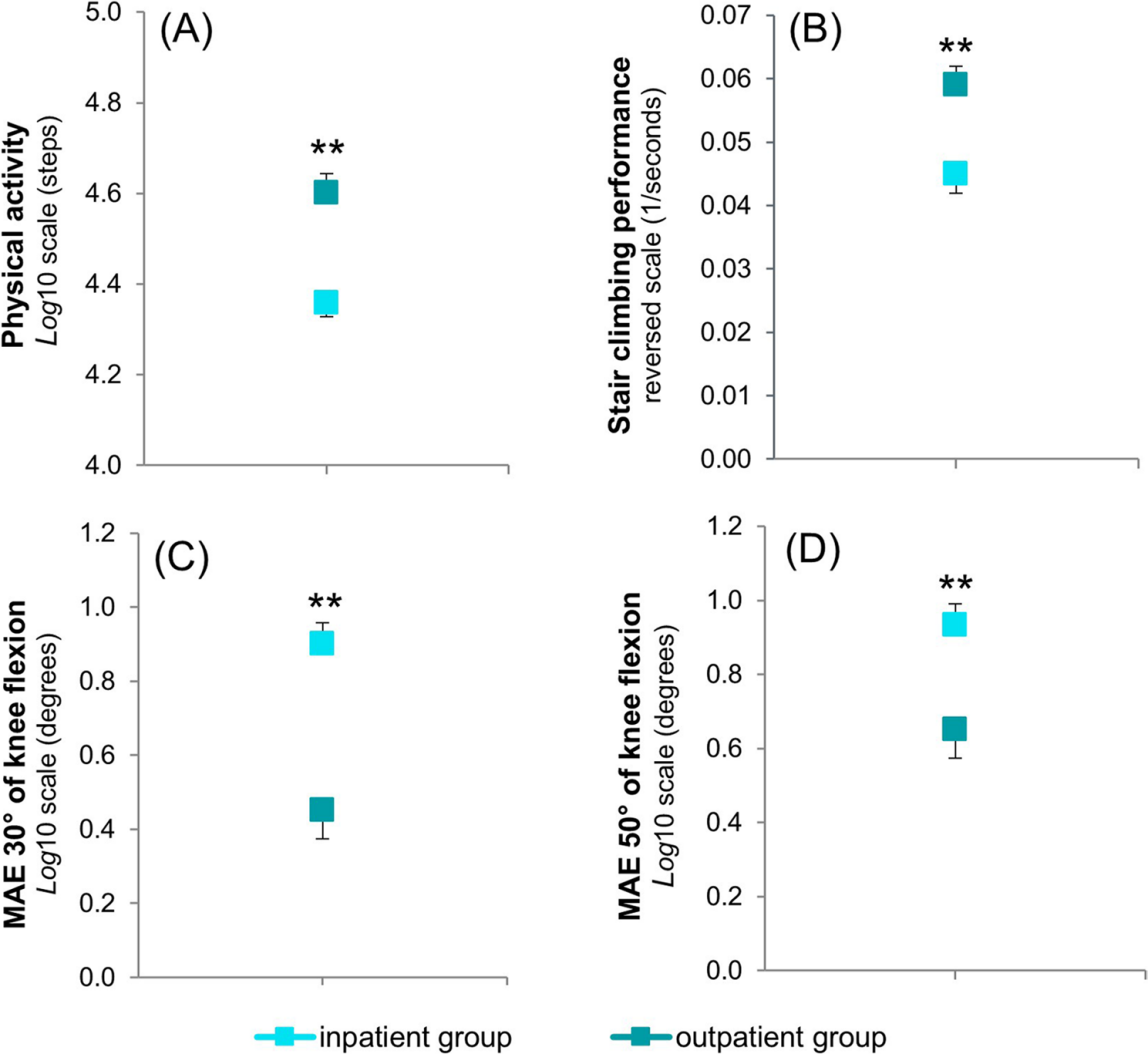
Results for (**A**) step count at pre-test (during the first 7 days of rehabilitation), (**B**) stair climbing performance at post-test, and mean absolute error (MAE) during the knee joint repositioning test at (**C**) 30° and (**D**) 50° of knee flexion at post-test. Data in (**A**) were analysed using ANCOVA model 1 (adjusted for age, BMI, sex, hospital, and number of interventions). Data in (**B**), (**C**), and (**D**) were analysed using ANCOVA model 2 (adjusted for baseline, age, BMI, sex, hospital, and number of interventions). Values are presented as covariate-adjusted means and standard error of the mean (SEM) on the respective analysis scale: log-transformed (**A**, **C**, **D**) or reverse-transformed **(B**). On the reverse-transformed scale in panel B, a higher position indicates a faster performance (lower time). The corresponding back-transformed means for the inpatient and outpatient groups, respectively, were: (**A**) 22,909 vs. 39,811 steps (+ 73.8%), (**B**) 22.2 vs. 16.9 s (– 23.7%), (**C**) 7.9° vs. 2.8° (–6 4.6%), and (**D**) 8.5° vs. 4.5° (– 47.1%). ** denotes a significant difference between groups (*P* ≤ 0.010)

The outpatient group had a significantly higher step count (+ 73.8%; large effect) at pre-test during the first 7 days of rehabilitation (Table 3; Fig. 3). No significant differences in step count were found at post-test, although the number of steps appeared to be slightly higher in the outpatient group (Table 3).

**Table 3:** Primary and secondary functional outcomes. Descriptive statistics are presented as raw means (standard deviations) on the original scale for both pre-test and post-test. Inferential statistics (adjusted mean differences, 95% CI) were derived from ANCOVA Model 1 (adjusted for age, BMI, sex, hospital, and number of interventions) or, for the total number of steps analysed, from an unpaired Student’s t-test. Results are reported on the scale indicated in the sStatistical test column. Statistical values (*P*,* F* and *η*_*p*_^*2*^*)* were calculated from original data or, in the case of non-normal distributions, from log-transformed (Log10) data. Note: Gait performance was assessed exclusively at post-test, as the use of walking aids at pre-test precluded valid spatio-temporal measurements

	Descriptive statistics: mean (standard deviation)				Inferential statistics						
	Inpatient group (*n* = 26)		Outpatient group (*n* = 16)		Statistical test	Time point of analysis	Adjusted mean difference (95% CI)	*P*	F	Effect size (η_*p*_^2^)	Power (β)
	Pre-test	Post-test	Pre-test	Post-test							
PRIMARY FUNCTIONAL OUTCOME											
**Step count** (n) for 7 days	26,345 (12603)	34,791 (15435)	37,704 (7882)	40,183 (14301)	Log10 / ANCOVA	Pre-test	-0.24 (-0.36 to -0.13)	< 0.001**	17.857	**0.338**	0.994
					Original /ANCOVA	Post-test	6655 (-4436 to 17747)	0.231	1.484	0.041	0.256
SECONDARY FUNCTIONAL OUTCOMES											
Sit-to-stand transitions (n) for 7 days	383.54 (107.22)	355.77 (81.97)	465.13 (122.48)	391.81 (111.1)	Original / ANCOVA	Pre-test	74 (-11 to 158)	0.086	3.117	0.082	0.470
					Original / ANCOVA	Post-test	16 (-55 to 87)	0.652	0.207	0.006	0.078
Gait performance											
Total number of steps analysed	-	36.92 (5.75)	-	36.25 (6.07)	Unpaired Student’s t-test.	Post-test	-0.67 (-4.44 to 3.10)	0.720	-	-	-
Gait velocity/height	-	0.69 (0.09)	-	0.70 (0.11)	Original / ANCOVA	Post-test	-0.01 (-0.08 to 0.06)	0.787	0.074	0.002	0.060
Stride length/height	-	74.72 (8.09)	-	75.31 (9.03)	Original / ANCOVA	Post-test	-1.46 (-7.01 to 4.09)	0.598	0.284	0.008	0.087
CV_Stride length_ (%)	-	3.18 (2.03)	-	3.17 (1.46)	Log10 / ANCOVA	Post-test	0.04 (-0.10 to 0.18)	0.602	0.277	0.008	0.087
Stance time (% gait cycle)	-	64.54 (1.98)	-	64.07 (1.44)	Original / ANCOVA	Post-test	-0.11 (-1.29 to 1.07)	0.851	0.036	0.001	0.055
Swing time (% gait cycle)	-	35.54 (2.02)	-	35.97 (1.40)	Original / ANCOVA	Post-test	0.13 (-1.07 to 1.34)	0.826	0.049	0.001	0.055
Single support time (% gait cycle)	-	35.59 (2.01)	-	36.06 (1.35)	Original / ANCOVA	Post-test	-0.05 (-1.23 to 1.12)	0.927	0.008	< 0.001	< 0.055
Total double support time (% gait cycle)	-	28.93 (3.93)	-	28.03 (2.75)	Original / ANCOVA	Post-test	< -0.01 (-2.31 to 2.31)	0.998	< 0.001	< 0.001	< 0.055

*CV*: Coefficient of variation, *95% CI*: 95% confidence interval, *P*: Probability value (** *P* ≤ 0.010), *F*: Critical F value of the F-distribution (variance of the group means / mean of the within group variances), Effect size (η_p_^2^): partial eta-squared, η_p_^2^ ≥ 0.01 small effect, η_p_^2^ ≥ 0.06 medium effect, η_p_^2^ ≥ 0.14 large effect indicated by bold text, Power: β < 0.50 low power, β ≥ 0.50 medium power, β ≥ 0.80 high power

### Secondary clinical and functional outcomes

At the post-test, the outpatient group showed significantly greater proprioceptive accuracy, with a lower mean absolute error during the knee joint repositioning test at both 30° (– 64.6%; large effect) and 50° (– 47.1%; large effect) of knee flexion (Table 4; Fig. 3). Furthermore, patients in the outpatient group were significantly faster at completing the stair climbing test (– 23.7%; large effect) (Table 4; Fig. 3). These significant findings were associated with high post-hoc power (*β* > 0.80).

**Table 4:** Secondary clinical and functional outcomes. Descriptive statistics are presented as raw means (standard deviations) on the original scale for both pre-test and post-test. Inferential statistics (adjusted mean differences, 95% CI) were derived from ANCOVA with baseline-adjustment model 2 (adjusted for pre-test, age, BMI, sex, hospital, and number of interventions) and are reported on the scale indicated in the statistical test column. Statistical values (*P*,* F* and *η*_*p*_^*2*^*)* were calculated from original data or, in the case of non-normal distributions, from log-transformed (Log10) or reverse-transformed (Reverse) data

	Descriptive statistics: mean (standard deviation)				Inferential statistics					
	Inpatient group (*n* = 26)		Outpatient group (*n* = 16)		Statistical test	Adjusted mean difference (95% CI)	*P*	F	Effect size (η_*p*_^2^)	Power (β)
	Pre-test	Post-test	Pre-test	Post-test						
SECONDARY CLINICAL OUTCOMES										
**Knee pain**	2.73 (1.90)	0.95 (1.24)	3.52 (1.81)	0.77 (1.39)	Log10 / ANCOVA	0.10 (-0.06 to 0.26)	0.210	1.634	0.046	0.283
**Knee joint swelling** (cm)	47.13 (3.10)	44.61 (2.46)	48.03 (4.94)	45.25 (5.10)	Log10 / ANCOVA	0.04 (-0.01 to 0.15)	0.536	0.385	0.011	0.101
**Knee joint range of motion** (°)										
Active knee flexion	87.44 (9.54)	111.05 (13.35)	85.08 (8.66)	112.09 (11.54)	Original / ANCOVA	2.19 (-6.46 to 10.84)	0.611	0.264	0.008	0.087
Passive knee flexion	92.66 (10.41)	114.95 (15.42)	90.46 (6.50)	116.41 (11.16)	Original / ANCOVA	2.46 (-6.78 to 11.70)	0.592	0.292	0.009	0.092
Active knee extension	6.64 (5.52)	4.88 (5.23)	9.49 (5.27)	5.41 (2.96)	Original / ANCOVA	-1.33 (-3.22 to 0.57)	0.164	2.022	0.056	0.336
Passive knee extension	5.12 (4.72)	3.88 (5.01)	7.20 (4.29)	3.86 (2.33)	Original / ANCOVA	-1.61 (-3.62 to 0.39)	0.111	2.673	0.073	0.424
SECONDARY FUNCTIONAL OUTCOMES										
**Timed up and go performance** (s)	18.42 (9.37)	9.48 (4.18)	22.21 (13.18)	9.21 (2.75)	Log10 / ANCOVA	0.03 (-0.05 to 0.10)	0.489	0.490	0.014	0.117
**Stair climbing performance** (s)	61.63 (33.34)	25.83 (12.69)	63.55 (23.78)	21.47 (10.27)	Reverse / ANCOVA	-0.01 (-0.02 to -0.01)	0.004**	9.474	**0.218**	0.914
**Joint position sense** (°)										
MAE 50° knee flexion	10.56 (4.88)	11.09 (9.22)	11.07 (5.74)	5.67 (4.64)	Log10 / ANCOVA	0.28 (0.07 to 0.49)	0.010**	7.455	**0.180**	0.839
MAE 30° knee flexion	6.64 (3.61)	10.32 (8.27)	7.02 (3.99)	3.37 (2.01)	Log10 / ANCOVA	0.45 (0.24 to 0.66)	< 0.001**	19.63	**0.366**	0.998
**Long-term memory representation of the gait**	(*n* = 25)		(*n* = 12)							
Lambda	0.71 (0.20)	0.66 (0.16)	0.61 (0.15)	0.62 (0.15)	Reverse / ANCOVA	-0.14 (-0.44 to 0.17)	0.364	0.851	0.028	0.188

*MAE*: Mean absolute error, *CV*: Coefficient of variation, *95% CI*: 95% confidence interval, *P:* Probability value (** *P* ≤ 0.010), *F*: Critical F value of the F-distribution (variance of the group means / mean of the within group variances), Effect size (η_p_^2^): partial eta-squared, η_p_^2^ ≥ 0.01 small effect, η_p_^2^ ≥ 0.06 medium effect, η_p_^2^ ≥ 0.14 large effect indicated by bold text, Power: β < 0.50 low power, β ≥ 0.50 medium power, β ≥ 0.80 high power

No significant between-group differences were found at the post-test for any other secondary outcomes, including knee pain, knee joint swelling, active and passive maximal knee flexion, and knee extension range of motion, timed up and go performance, any of the measured spatio-temporal or temporophasic gait parameters, or long-term memory representation of the gait. These non-significant findings were associated with low post-hoc power (*β* < 0.50) (Tables 3 and 4).

### Correlations between outcomes

The step count was strongly and positively correlated with the number of sit-to-stand transitions (*P* = 0.003) at post-test. Furthermore, stair climbing and timed up and go performance showed a strong positive correlation (*P* < 0.001). No other statistically significant correlations were found (Table 5).

**Table 5:** Correlation matrix of clinical and functional outcomes at post-test. Partial correlation analyses (Spearman’s rho) adjusted for age, BMI, sex, hospital, and number of interventions during rehabilitation. Correlations were calculated using the pooled sample (*n* = 42) including both inpatient and outpatient groups. Exploratory within-group analyses indicated that correlation patterns were generally consistent across both groups

	Step count	Sit-to-stand transitions	Timed up and go performance	Stair climbing performance	Knee pain	Active knee flexion ROM
Step count	1					
Sit-to-stand transitions	0.482**	1				
Timed up and go performance	-0.191	-0.049	1			
Stair climbing performance	-0.309	-0.051	0.676**	1		
Knee pain	-0.295	0.123	0.203	0.124	1	
Active knee flexion range of motion	0.128	0.168	-0.247	-0.217	-0.294	1

:Spearman correlation coefficient: |r_s_| ≥ 0.1 weak correlation, |r_s_| ≥ 0.3 moderate correlation, |r_s_| ≥ 0.5 strong correlation indicated by bold text, ** *P* ≤ 0.010 indicated by bold text

## Discussion

This study is the first comparing the effects of inpatient versus outpatient rehabilitation following TKA on clinical and functional outcomes within the unique context of the German healthcare system. Compared with international norms, this system is characterized by longer hospital stays followed by more intensive, standardized rehabilitation protocols.

Previous studies conducted in Germany have indicated that orthopaedic outpatient rehabilitation offers greater cost-effectiveness [9, 10, 15] and comparable quality compared to inpatient care, showing no differences across a wide range of clinical, functional, and safety outcomes [9–14]. A commonality among these studies is that effectiveness was evaluated for mixed degenerative musculoskeletal conditions (e.g., end-stage KOA, degenerative spine, other joint replacements, chronic pain) [9–14, 26], but did not focus specifically on patients with TKA.

The primary finding of this study is that the outpatient group was significantly more active, demonstrating a higher step count during the first week of rehabilitation. Patients who self-selected outpatient care are likely inherently more motivated as well as mobile, and the home setting imposes a continuous functional demand (e.g., engaging in instrumental activities of daily living such as shopping or housekeeping). Since increased physical activity is strongly associated with improved functional outcomes after TKA [27, 28], the physical requirements of living at home may act as a powerful stimulus for recovery. This early physical activity appears to be of high relevance, as indicated by the positive correlations between step count and the number of sit-to-stand transitions, as well as the negative association with the pain level of patients. Physiologically, high levels of early activity act as a recovery stimulus by providing consistent afferent feedback to the central nervous system, which likely served as a trigger signal for neuroplastic reorganization and helped to prevent the progression of chronic muscle inhibition [29, 30].

Arthrogenic muscle inhibition (AMI) is a physiological phenomenon characterized by the inability to fully activate muscles voluntarily following joint surgery or trauma [31]. In the context of TKA, AMI represents as a neural inhibition of the quadriceps muscle, which is triggered by pain, swelling, and the operative removal of joint mechanoreceptors. This inhibition leads to a profound loss of muscle strength, often exceeding 60% in the first postoperative month, which contributes significantly to functional limitations and increased fall risk [32]. While quadriceps muscle activation failure can cause a 90% decline in stair climbing performance immediately after surgery [32], early physical activity, such as walking and stair climbing, is essential for counteracting the effects of AMI [33].

Consequently, the better stair climbing performance and joint position sense observed in the outpatient group at post-test further reinforce the conclusion that superior functional outcomes are driven by the intensity and frequency of physical activity required in the home setting. These findings are highly relevant given that the ability to ascend and descend stairs confidently is a key determinant of a patient’s independence at home.

The large effect sizes and high power (*β* ≥ 0.80) of these significant results (step count during the first week of rehabilitation, joint position sense, and stair climbing performance) confirm the robustness of these differences. Conversely, the interpretation of secondary outcomes (knee pain, knee joint swelling, range of motion, timed up and go performance, gait performance, and long-term memory representation of the gait) must consider the small-to-medium effect sizes and observed low power (*β* < 0.50), which indicates a high risk of type II error due to the small sample size.

These findings suggest that both inpatient and outpatient rehabilitation in the German health care system provide sufficient therapeutic efficiency to restore basic mobility and function to a comparable level. However, the superior results for more demanding tasks in the outpatient group, suggest that this rehabilitation model might prepare patients better for the challenges of daily life.

A growing body of evidence has shown comparable outcomes for patients with TKA between inpatient and outpatient rehabilitation in other countries [3–6]. The present study adds to this consensus by demonstrating its applicability within the highly structured German healthcare system. The finding that outpatients achieved better outcomes in specific functional domains, despite receiving a similar overall volume of therapeutic interventions (Table 3), suggests that the context of rehabilitation (inpatient vs. outpatient) is a crucial variable. The outpatient rehabilitation pathway is suitable for patients who are more mobile, motivated, and have a stable and supportive home environment. In contrast, the inpatient pathway remains recommended for patients with a more complex medical history, significant comorbidities, or those who lack adequate support in their home environment.

### Study limitations

The failure to reach the target sample size for the outpatient group resulted in low statistical power (*β* < 0.50) for several secondary outcomes, increasing the risk of type II errors. Although we statistically controlled for baseline imbalances using hospital characteristics and intervention volume as covariates, these adjustments cannot fully compensate for the lack of randomization. Additionally, the non-randomized design introduces a significant risk of selection bias, i.e., patients opting for outpatient care may have been inherently more motivated, healthier, or had better social support than those opting for inpatient care.

While the activPAL™ sensor placement transitioned from the unaffected limb at pre-test to a standardized placement at post-test, the high test-retest reliability and side-invariance of the device, combined with the typical stabilization of gait patterns by 13 weeks post-TKA [16, 18], suggest that this approach minimized rather than introduced measurement bias.

A further limitation of the current study is the focus on clinical and objective functional parameters. Therefore, future research should include patient-reported outcome measures to provide a more holistic view of the patient’s recovery.

## Conclusion

Our preliminary findings indicate that outpatient rehabilitation following TKA in the German healthcare system is associated with higher early physical activity levels (i.e., step count during the first 7 days of rehabilitation) and superior outcomes in specific functional tasks, such as stair climbing and joint position sense at 3 months post-operatively. These results suggest that the home environment may provide a relevant functional stimulus for recovery. However, due to the non-randomized, preference-based study design and the small sample size, these results must be interpreted with caution, as selection bias and residual confounding cannot be excluded.

While many clinical outcomes showed no significant between-group differences, the low statistical power for these measures necessitates further research with larger cohorts to identify potential clinically meaningful effects. Outpatient rehabilitation appears to be a viable option for motivated patients with a supportive home environment, aligning with international trends toward ambulatory care. Future multicenter randomized controlled trials are required to confirm and extent these findings helping to establish evidence-based criteria for optimal post-TKA recovery pathways.

## Supplementary Information

Supplementary Material 1.

## Data Availability

The datasets used and/or analysed during the current study are available from the corresponding author on reasonable request.

## Abbreviations

AMI: Arthrogenic muscle inhibition
ANCOVA: Analysis of covariance
BAC: basic action concept
BMI: body mass index
CI: Confidence interval
CV: Coefficient of variation
ICD: International Statistical Classification of Diseases and Related Health Problems
KOA: knee ostheoarthritis
MAE: Mean absolute error
MD: mean difference
SDA-M: Structural dimensional analysis of mental representation
TKA: Total knee arthroplasty
