# Supplementary material for: Effectiveness of inpatient versus outpatient rehabilitation following total knee arthroplasty on clinical and functional outcomes in Germany: a non-randomized clinical trial
Source: BMC Sports Sci Med Rehabil. 2026 May 23;18:248. doi: 10.1186/s13102-026-01735-4 (PMC13200373; doi:10.1186/s13102-026-01735-4)
Supplement: Supplementary file 1 — Supplementary Material 1. [file 13102_2026_1735_MOESM1_ESM.docx]

#### Reliability of Knee Joint Swelling and Range of Motion Measurement

Eleven patients with KOA (nine females, two males; age: 67.0 ± 7.9 yrs; height: 1.62 ± 0.09 m; weight: 91.6 ± 11.8 kg) participated in one experimental session. Knee joint circumference and knee flexion ROM were measured as described above. Two measurements were performed and intra-rater reliability between these two trials was calculated using an Excel spreadsheet developed by Hopkins [1]. The typical error of measurement and CV were calculated to provide measures of absolute reliability [2]. Relative reliability was estimated using the ICC with 95% confidence interval [2]. ICC and CV values were classified as follows: CV < 10% and ICC > 0.90 were considered high; CV between 10%–20% and ICC between 0.80–0.90, moderate; and CV > 20% and ICC < 0.80, low reliability [3,4]. Between-trial differences were assessed using paired Student’s *t* tests with the SPSS statistical package (version 22.0; SPSS Inc., Chicago, IL, USA). The significance level was set at *P*≤ 0.050.

Statistical analyses showed no significant between-trial differences. Absolute and relative intra-session reliability for knee joint circumference and active and passive maximal knee flexion ROM was very high (CV values < 5.89%; ICC values = 0.99) indicating excellent repeatability of measurements (see Additional file table 1).

**Additional file table 1.** Intra-session reliability of knee joint circumference and knee flexion range of motion measurement.

|  | Trial 1  Mean (SD) | Trial 2  Mean (SD) | Mean difference  (95% CI) | SD_Diff_ | TE  (95% CI) | CV  (95% CI) | ICC  (95% CI) |
| --- | --- | --- | --- | --- | --- | --- | --- |
|  |  |  |  |  |  |  |  |
| **Knee circumference** | **cm** | **cm** | **cm** | **cm** | **cm** | **%** |  |
|  | 6.82 (3.07) | 7.08 (3.15) | 0.26 (-0.10 to 0.63) | 0.54 | 0.14 (0.10 to 0.25) | 5.89 (4.08 to 10.56) | 0.99 (0.95 to 1.00) |
|  |  |  |  |  |  |  |  |
| **Range of motion** | **deg** | **deg** | **deg** | **deg** | **deg** | **%** |  |
| Active KF | 114.25 (8.83) | 114.61 (8.11) | 0.35 (-0.38 to 1.09) | 1.10 | 0.10 (0.07 to 0.18) | 0.81 (0.56 to 1.42) | 0.99 (0.97 to 1.00) |
| Passive KF | 118.45 (9.21) | 118.65 (8.50) | 0.21 (-0.64 to 1.06) | 1.27 | 0.11 (0.07 to 0.19) | 0.83 (0.58 to 1.46) | 0.99 (0.97 to 1.00) |

Abbreviations: SD, standard deviation; CI, confidence interval; SD_Diff_, SD of the difference between trials 1 and 2; TE, typical error; CV, coefficient of variation; ICC, intraclass correlation coefficient; KF, knee flexion; KE, knee extension.

# **References**

1. Hopkins WG: **Precision of measurement. In: A New View of Statistics** [http://sportsci.org/resource/stats/precision.html].

2. Atkinson G, Nevill AM: **Statistical methods for assessing measurement error (reliability) in variables relevant to sports medicine**. *Sports medicine (Auckland, N.Z.)* 1998, **26**:217-238.

3. Vincent WJ: *Statistics in kinesiology*. 4th edition. Champaign, Ill.: Human Kinetics; 2012.

4. Stokes M: **Reliability and Repeatability of Methods for Measuring Muscle in Physiotherapy**. *Physiotherapy Practice* 1985, **1**:71-76.
